# Correlation Between Regulation of Intestinal Flora by Danggui‐Shaoyao‐San and Improvement of Cognitive Impairment in Mice With Alzheimer's Disease

**DOI:** 10.1002/brb3.70110

**Published:** 2024-10-31

**Authors:** Ya‐Han Wang, Peng‐Li Ding, Kai‐Xin Zhang, Xiang‐Qing Xu, He Li

**Affiliations:** ^1^ Department of Neurology Affiliated Hospital of Shandong University of Traditional Chinese Medicine Jinan China; ^2^ The First Clinical Medical College Shandong University of Traditional Chinese Medicine Jinan China

**Keywords:** Alzheimer's disease, central glucose metabolism, Danggui‐Shaoyao‐San, intestinal homeostasis

## Abstract

**Purpose:**

The abnormal central glucose metabolism in Alzheimer's disease (AD) is related to the brain–gut axis. This study aims to explore the target of Danggui‐Shaoyao‐San (DSS) in improving cognitive impairment.

**Method:**

This study analyzed the differences in mice intestinal flora by 16S rRNA sequencing. The cognitive protective effects of DSS were observed through the Morris water maze and the new object recognition. The mitigation effects of DSS on Aβ and p‐tau, regulatory effects on glucose metabolism targets, and intestinal structure effects were observed through brain and colon slices staining. The differences in neural ultrastructure were compared by transmission electron microscopy.

**Finding:**

The results showed that DSS affected the composition of intestinal dominant bacteria and bacteria genera and regulated the abundance of intestinal bacteria in AD mice. DSS improved the behavior of AD mice, alleviated the deposition of AD pathological products in the brain and colon, regulated the expression of glycometabolism‐related proteins, and improved the colon barrier structure and neural ultrastructure in the brain of mice with AD.

**Conclusion:**

Our findings suggest that DSS may affect AD central glucose metabolism and improve cognition by regulating the gut–brain axis.

## Graphical abstract

DSS regulated the gut microbiota of AD mice, improved the cognition of AD mice, reduced the deposition of pathological products of AD in the brain and colon, regulated the expression of proteins related to glucose metabolism, and improved the brain–colon barrier structure and neural ultrastructure of AD mice.

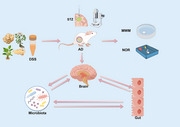

## 1 Introduction

Alzheimer's disease (AD) includes familial AD (FAD), which is strongly associated with genetic mutations, and late‐onset sporadic AD (SAD), which is influenced by genetic, lifestyle, and environmental factors. SAD, also known as Type 3 diabetes, is characterized by central insulin resistance and unaffected peripheral glucose metabolism. This central glucose metabolism abnormality seems to be associated with intestinal microbial homeostasis through the gut–brain axis, and the two interact to affect the onset of AD (Cowan and Cryan 2021).

A gut–brain axis regulation system has been proposed based on the bidirectional regulation of the gut microbiota and the central nervous system. All bacteria, viruses, fungi, and archaea in the human gut are collectively known as the gut microbiome, which plays key roles in neurodevelopment, cognition, and behavior (Ma et al. 2019). In preclinical and clinical studies of AD, dysregulation of the gut microbiota, caused by changes in the diversity and frequency of microbial groups and species residing in the gut, has been associated with excessive amyloid deposition, inflammation, immunity, and damage to neurons and synapses (Cuervo‐Zanatta, Garcia‐Mena, and Perez‐Cruz 2021; Govindarajulu et al. 2020). The metabolic characteristics of some intestinal microorganisms have become research hotspots in AD (Rawan and Elena 2022).

Danggui‐Shaoyao‐San (DSS) is a classic Chinese medicinal compound known as Toki‐shakuyaku‐san in Japan and Dangguijakyakan in Korea. It comes from the ancient Chinese medicine book Jinguiokiluo. There is evidence of the clinical efficacy of DSS in the treatment of dementia (Berk and Sabbagh 2013; K. H. Kim et al. 2016; Kitabayashi et al. 2007; Matsuoka et al. 2012; Park et al. 2012). Preclinical trials have also found that DSS can have neuroprotective effects by alleviating amyloid neurotoxicity, promoting long‐term enhancement, clearing the free radical self, and regulating neurotransmitter release (Ding et al. 2018; Hu et al. 2012; Watanabe et al. 1995; Wu et al. 2020; Zhang, Hagino, and Nozaki 1997). In a study on metabolic syndrome, DSS improved blood sugar and lipid metabolism in rats by regulating the gut microbiota (Yin et al. 2021). The purpose of the present study was to explore whether DSS could regulate the intestinal flora in AD model mice and to determine the correlation between changes in this flora and cognitive function.

## 2 Methods

### 2.1 Animals

Six‐month‐old male C57/BL6J mice (Beijing Vital River Laboratory Animal Technology Co. Ltd., Beijing, China; license number [SCXK(Zhejiang)2021‐0006]) were housed in the SPF Animal Feeding Room (SYXK [Lu] 20220009), maintained at controlled conditions (20–22°C, relative humidity 60%–70%, and 12‐h light/dark cycle). Thirty mice were intracerebroventricularly (icv) injected with the neurotoxin streptozotocin (STZ) to simulate AD development. This molding method is consistent with our previous research (Qin et al. 2021). Fifteen mice were injected with an equal volume of artificial cerebrospinal fluid in the lateral ventricle as the control group. The Animal Ethics Committee of the Shandong University of Chinese Medicine approved the experiment (SDUTCM20230407002), and all experiments complied with ARRIVE guidelines.

### 2.2 Drug Treatment

The daily dose of DSS for human use includes Danggui (9 g), Shaoyao (48 g), Chuanxiong (10 g), Poria (12 g), Zexie (24 g), and Baishu (12 g). The daily dose of each mouse = human dose (g) /60 kg × 9.1 × 0.03. Fifteen AD mice in the DSS group were treated with DSS (daily dose for 15 mice: Danggui 0.6 g, Shaoyao 3.2 g, Zexie 1.6 g, Baizhu 0.8 g, Poria 0.8 g, and Chuanxiong 0.7 g) for 30 days (0.1 mL/10 g BW). DSS non‐decocting granules (batch number: 2306500101) were dissolved in double steaming water (the volume of double steaming water was calculated according to 0.1 mL/10 g BW), preserved at 4°C and administered once a day. We ensured that the DSS granules were fully dissolved by heating and stirring and shook up and down 5 times before each intragastric administration to mix thoroughly. Equal volumes of distilled water were administered to the control and AD groups.

### 2.3 16S rRNA Sequencing

After 30 days of intervention, the feces of each group of mice were collected. This sequencing process is consistent with previous studies (Qiu et al. 2022).

### 2.4 Morris Water Maze

The arrangement and implementation of the swimming pool were consistent with previous studies (Qin et al. 2021). Swimming distance, time taken to find the platform after entering the water from the second quadrant (training and testing periods), and time spent in the first quadrant was recorded (after removing the platform).

### 2.5 New Object Recognition

Cognitive performance was measured based on the number, time, and distance at which the mice explored new and old objects. The implementation process was consistent with previous studies (Owen et al. 2022). The recognition index (RI) was determined using Equation (1):

(1) $$\mathrm{RI}=\mathrm{new}\;\mathrm{object}/\left(\mathrm{new}\;\mathrm{object}+\mathrm{old}\;\mathrm{object}\right)\times 100$$

### 2.6 Fresh Tissue Specimens

The mice were decapitated and killed after undergoing abdominal anesthesia (anesthetic: tribromoethanol [Avertin], 0.1 mL/10 g), and their brain and colon tissues were dissected on ice. The tissues placed in EP tubes were transferred to a liquid nitrogen tank for preservation (*n* = 10 samples per group).

### 2.7 Paraffin Sectioning

All the brains and colons were dehydrated, embedded in paraffin, and sectioned. The brains and colons were sliced sequentially (4‐µm thick). Four mice were used in each group.

### 2.8 Alcian Blue‐Periodic Acid–Schiff Staining

Fixed colons were embedded in paraffin (Swiss‐roll technique) and stained with hematoxylin and eosin. Alcian blue‐Periodic Acid–Schiff (AB‐PAS) staining was performed as previously described (Grabrucker et al. 2023).

### 2.9 Immunohistochemistry

The implementation process is consistent with the previous literature (Qin et al. 2021). The following antibodies were used in this study: anti‐beta‐amyloid (anti‐Aβ) 1–42 [mOC64] (1:1000, ab201060; Abcam, Cambridge, UK); anti‐tau (phospho S396) (1:1000, ab32057; Abcam); anti‐GSK3beta (1:5000, ab32391; Abcam); anti‐insulin receptor substrate 1 (IRS1) (1:1000, ab5603; Abcam); and animal non‐immune serum (sheep) (SP KIT‐B2; Fuzhou Maixin Biotech. Co. Ltd., Fuzhou, China). The stained areas in the thalamus and colon were measured using ImageJ software for each group of four samples.

### 2.10 Silver Plating Staining

The tissue slices were placed in xylene I for 10 min, xylene II for 10 min, xylene III for 10 min, anhydrous ethanol I for 5 min, anhydrous ethanol II for 5 min, 95% alcohol for 5 min, and 85% alcohol for 5 min and then washed in distilled water three to five times. Silver glycine dye solution C was added to the chemical ring, dyed for 5 min, and washed with distilled water three times; silver glycine dye solution B (preheated at 37°C) was added to the histochemical ring and treated for 3–5 min. The sections were removed, after which the residual silver glycine dye solution B was quickly shaken off, and they were placed into silver glycine dye solution A (preheated 15 min at 45°C) to observe the reduction effect. Next, the sections were removed after a few seconds, quickly shaken, and placed in fresh silver glycine dye solution A (preheated for 15 min at 45°C). After several seconds, the samples were removed and washed with distilled water. If the dyed background was too dark, it was treated with silver glycine dye solution D and washed three times with distilled water. The slices were dehydrated using anhydrous ethanol I for 5 min, anhydrous ethanol II for 5 min, anhydrous ethanol III for 5 min, dimethyl I for 5 min, and xylene II for 5 min and sealed with neutral gum. SlideViewer image analysis software was used to observe neurofibrillary tangles (NFTs) in each group (×20).

### 2.11 Western Blotting

The operation process is consistent with that reported in previous studies (Qin et al. 2021). Anti‐tau (phospho S396) antibody (1:1000, ab32057; Abcam) was used.

### 2.12 Transmission Electron Microscopy

After perfusion fixation was performed in the same method as in previous studies (Qin et al. 2021), the skulls of decapitated mice were fully exposed, which were opened using a bone rongeur. The brains were pulled apart in the sagittal position and placed on a glass plate. The brain was immediately placed in an EP tube containing 2.5% glutaraldehyde to stabilize the hippocampus. After fixation at 4°C for 2 h, the samples were cleaned using 0.1 MPB buffer for 10 min, which was repeated in triplicate and then preserved. The samples were then sent to the Shandong Aimeng Biotechnology Co. Ltd. (Jinan, China) for section preparation. The ultrastructure of neurons, synapses, mitochondria, and myelin was observed at ×20.0k and ×2.5k multifold (*n* = 3 samples per group).

### 2.13 Statistical Analysis

SPSS software (ver. 19.0; SPSS Inc., Chicago, IL, USA) and GraphPad Prism (ver. 9.4.1; GraphPad Software, La Jolla, CA, USA) were used to perform statistical analyses and data mapping. One‐way analysis of variance was used for intragroup data analysis. An independent sample *t*‐test was used for intergroup data analysis. Statistical significance was set at *p* < 0.05 and *p* < 0.01 denotes a higher significance level.

## 3 Results

### 3.1 Intestinal Flora 16S Sequencing

**FIGURE 1:**
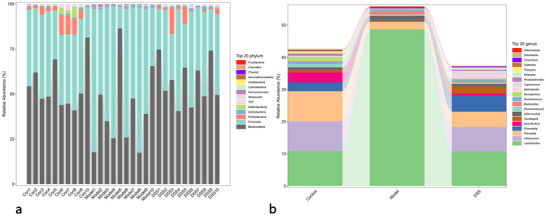
Analysis of relative species abundance at phyla and genus levels. The horizontal coordinate shows the three groups, the vertical coordinate shows the relative abundance percentages and different colors represent different phyla/genera. (a) Phylum‐level flora relative abundance analysis. (b) Genus‐level species relative abundance analysis.

The relative abundances of bacteria in each group at the phylum level are shown in Figure 1. Bacteroidetes (51%) and Firmicutes (40%) dominated intestinal flora. The total abundance of dominant bacteria accounted for > 90%, and the sum of the relative abundances of the dominant bacteria in all samples was 80%–95%. The ratio of Firmicutes to Bacteroidetes (F/B) in the AD group was higher than that in the control and DSS groups, suggesting that the composition of the dominant intestinal microphyla was significantly altered in AD, and the F/B ratio was increased. The species composition of the three groups of samples at the genus level was further investigated through species abundance analysis at the genus level (Figure 1), which showed that the intestinal environment was mainly dominated by *Lactobacillus* (24%), *Allobaculum* (6.7%), and *Prevotella* (6.7%). The proportion of *Lactobacillus* in the AD group was higher than that in the control and DSS groups, whereas the proportions of *Mycoplasma* and *Prevotella* were lower, suggesting a regulatory effect of DSS on intestinal flora.

**FIGURE 2:**
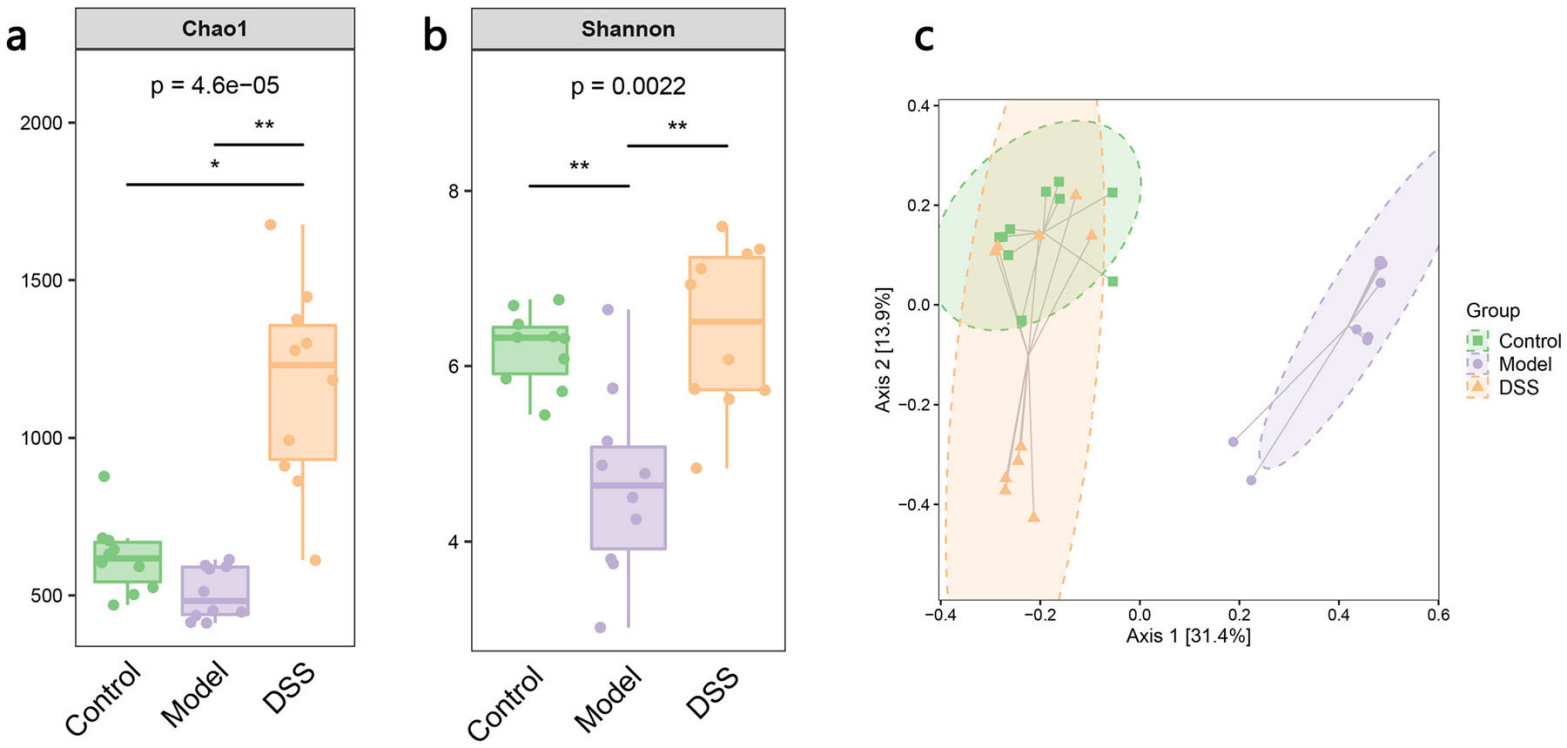
Alpha‐ and beta‐diversity analyses. (a) The larger the Chao1 index, the greater the number of species in the corresponding group. (b) The higher the Shannon index, the higher the alpha diversity. (c) The longer the horizontal coordinate distance, the greater the difference in community structure between groups, and the larger the overlap area between different groups indicates a more similar community composition structure (*p < 0.05, **p < 0.01).

In terms of community structure differences among the three groups, diversity analysis results showed that although there was no significant change in intestinal species abundance (chao1) in the AD group compared to that in the control group (Figure 2), the diversity of intestinal species composition decreased (Shannon; **p < 0.01) (Figure 2). Species abundance and diversity in the gut after DSS intervention were higher than those in the AD group (**p < 0.01) (Figure 2). Beta‐diversity analysis (Figure 2) showed that the community structures of the AD group and the other two groups were significantly separated on the *x*‐axis (PCoA1 = 31.4%), indicating that AD affected bacterial diversity and community composition. There was a partial overlap in beta diversity between the DSS and control groups, suggesting that the flora could recover in the direction of the normal population after DSS intervention. In addition, the results of the pairwise comparison of the Adonis multivariate displacement variance test further suggested differences in bacterial community structure among the different groups (Table 1). The difference between the AD and control groups was *F *= 12.785954 (*p* = 0.001), and that between the DSS group and AD group was *F *= 12.585036 (*p* = 0.001). In summary, the results showed that AD changed the species diversity of the gut, whereas DSS significantly improved species diversity and species richness. These results were highly consistent with those of the composition analysis, suggesting that DSS could improve the intestinal microbial composition of the host and largely restore it to a healthy state.

**TABLE 1:** Adonis multivariate permutation variance test for pairwise comparison.

		Sample size	Permutations	pseudo‐*F*	*p* value	*Q* value
Group1	Group2					
Control	DSS	20	999	3.157949	0.001	0.001
	AD	20	999	12.785954	0.001	0.001
DSS	AD	20	999	12.585036	0.001	0.001

**FIGURE 3:**
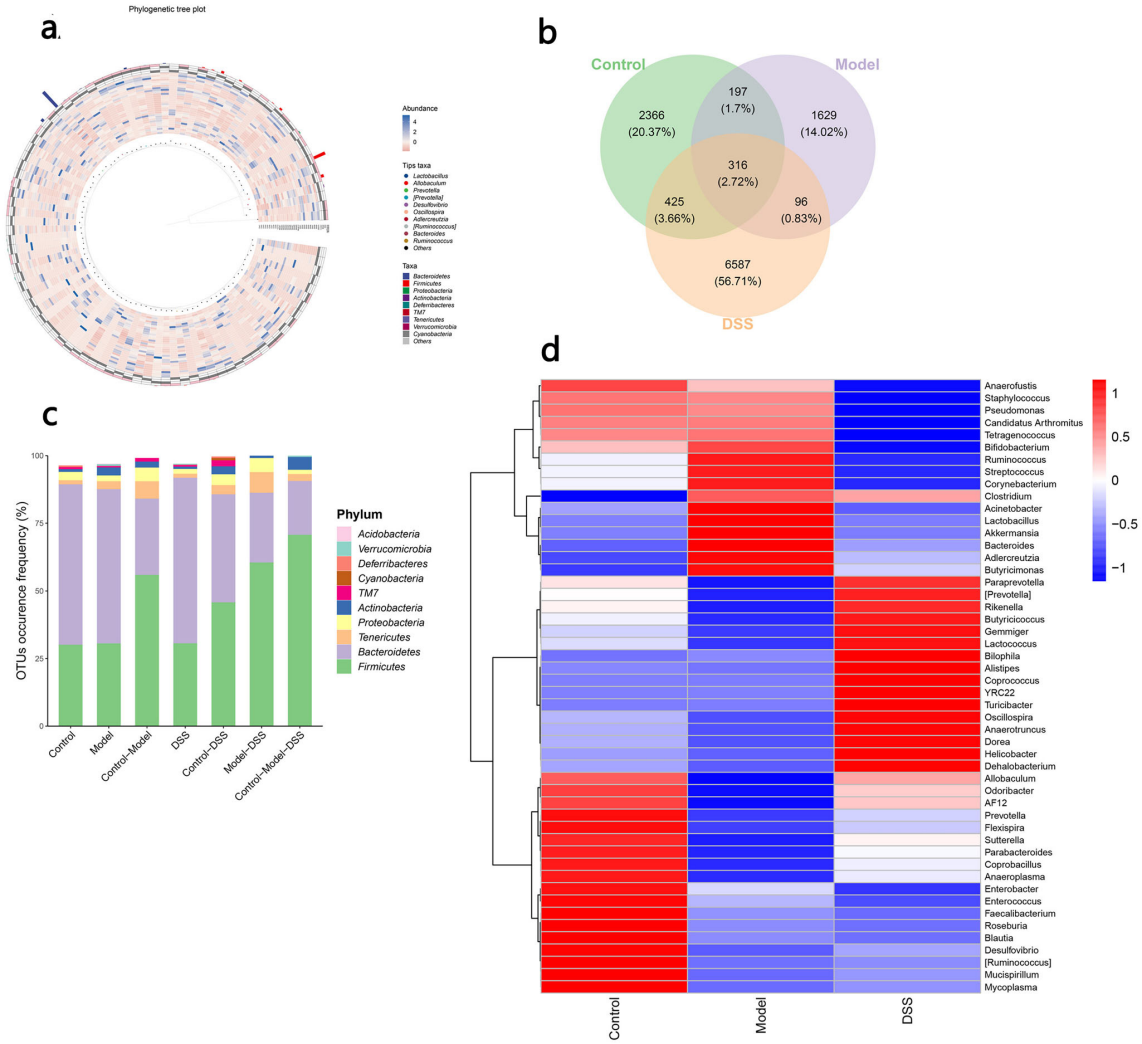
Difference analysis of microbial composition. (a) Top 100 core operational taxonomic units (OTUs). (b) Venn diagram analysis. Common and unique conditions of OTUs are shown in the three groups. (c) Occurrence frequency of common OTUs in the three groups. (d) Microbial abundance differences at the genus level.

The data analysis above investigated whether there were differences in the community composition and structure among the different experimental groups at the macro level. We further explored whether there were differences in specific microbial composition among the groups. The top 100 core operational taxonomic units (OTUs) in the intestines were mainly Bacteroidetes and Firmicutes (Figure 3). The distribution of core OTU abundances differed among the three groups. The Venn diagram results (Figure 3) showed common and unique OTUs in the control, AD, and DSS groups. There were 316 OTUs in the three groups, accounting for 2.72% (Firmicutes accounted for 60% and Bacteroidetes for 25%; Figure 3). There were 1629 OTUs unique to the AD group, accounting for 14.02% (Firmicutes accounted for 28% and Bacteroidetes for 59%; Figure 3). There were 6587 unique OTUs in the DSS group, accounting for 56.71% (Firmicutes accounted for 29% and Bacteroidetes for 62%; Figure 3). Figure 3 shows that there were significant differences in the abundance of microorganisms at the genus level among the three groups; 18 genera were significantly enriched in the control group, 11 were significantly enriched in the AD group, and 16 were significantly enriched in the DSS group.

**FIGURE 4:**
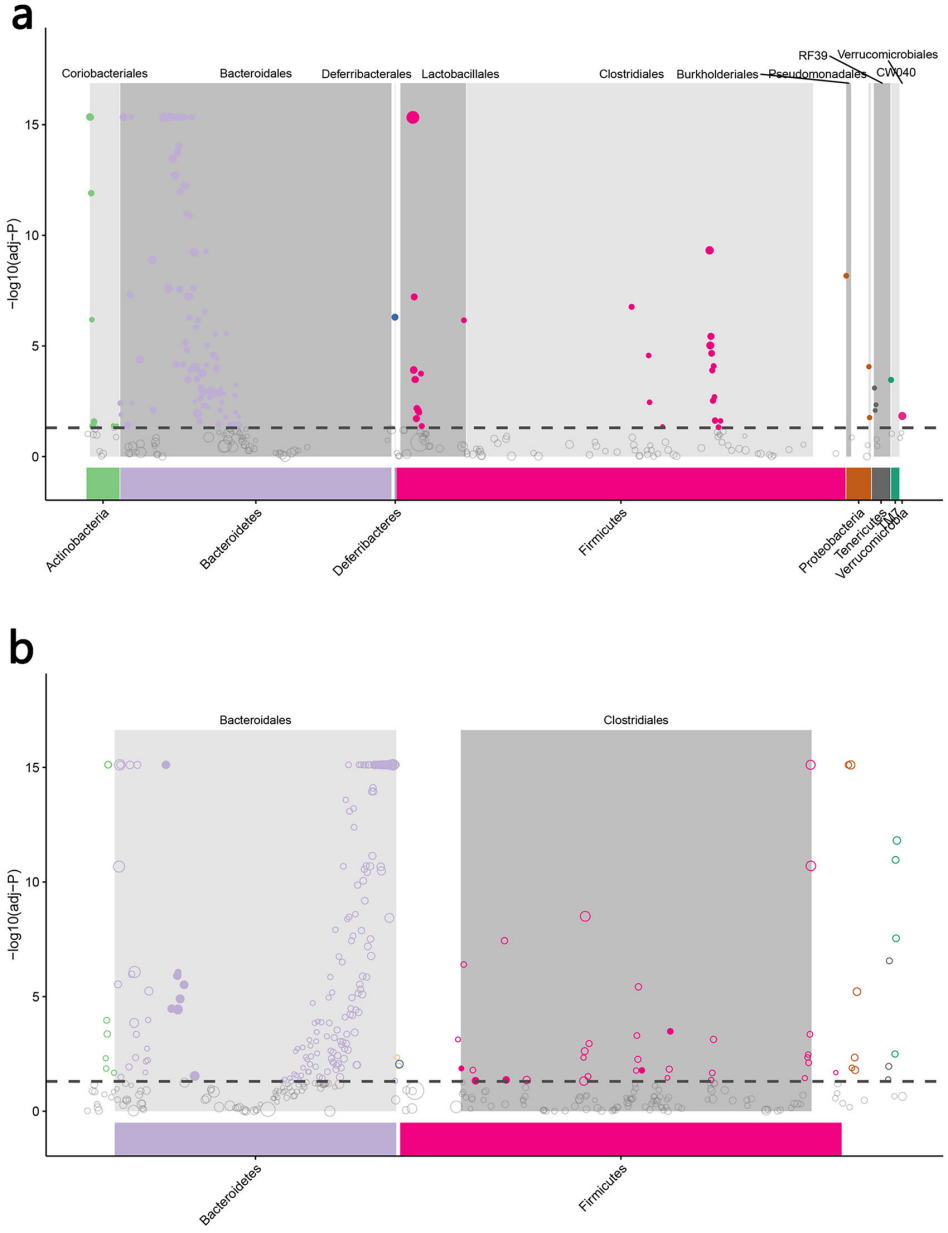
MetagenomeSeq difference analysis. (a) Differences of operational taxonomic unit (OTU) composition between control and Alzheimer's disease (AD) groups. (b) Differences of OTU composition between AD and Danggui‐Shaoyao‐San (DSS) groups.

MetagenomeSeq difference analysis was used to explore the differences in OTU composition among the different groups. Figure 4 shows that the AD group was significantly enriched in 163 OTUs compared to the control group, mainly belonging to metabacteria (Lactobacilli and *Burkholderia*) and Bacteroidetes (Bacteroidei). Among them, *Lactobacillus* is an important component of intestinal probiotics, which indicates that after the host is ill, it may initiate a regulatory mechanism, making the host instinctively respond to selectively enriched probiotics that are beneficial to their own health, to restore host intestinal homeostasis. Compared to the AD group, the DSS group was significantly enriched in 188 OTUs (Figure 4), which mainly belonged to Opicutes (Clostridiales) and Bacteroidetes (Bacteroideales). Some Clostridiales have anti‐inflammatory effects and maintain intestinal health through the synthesis of butyrate metabolites (Kropp et al. 2021), and DSS intervention may accelerate the progress of host rapid selection of beneficial microorganisms (such as anti‐inflammatory microorganisms) to promote the improvement of host intestinal microbes.

**FIGURE 5:**
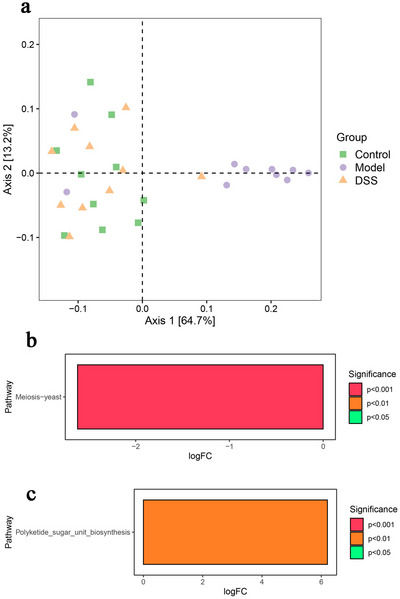
Predictive analysis of community function. (a) The farther away the X wheelbase, the greater the difference in community function. (b) Compared to the control group, Alzheimer's disease (AD) group was enriched in meiosis−yeast function (*p < 0.05). (c) Compared to the AD group, polyketide sugar unit biosynthesis function was enriched in Danggui‐Shaoyao‐San (DSS) group (*p < 0.05).

The predictive analysis of community function using Picrust software showed that the AD group was significantly separated (PCoA1 = 64.7%) on the *x*‐axis (the main influencing factor), whereas the control and DSS groups showed no significant difference in functional composition (Figure 5). Compared to the control group, the AD group was enriched in meiosis–yeast function (*p* < 0.05) (Figure 5), which is mainly involved in cell reproduction, suggesting that the selection mechanism of the host could be activated to accelerate the increase in microbial flora after disease. Compared to the AD group, the polyketide sugar unit biosynthesis function was enriched in the DSS group (*p* < 0.05) (Figure 5), which is conducive to intestinal probiotics, inhibited the growth and reproduction of pathogenic microorganisms, and promoted intestinal homeostasis.

### 3.2 DSS Improves Cognitive Impairment and Pathological Changes in Central Nervous System of Mice With AD

**FIGURE 6:**
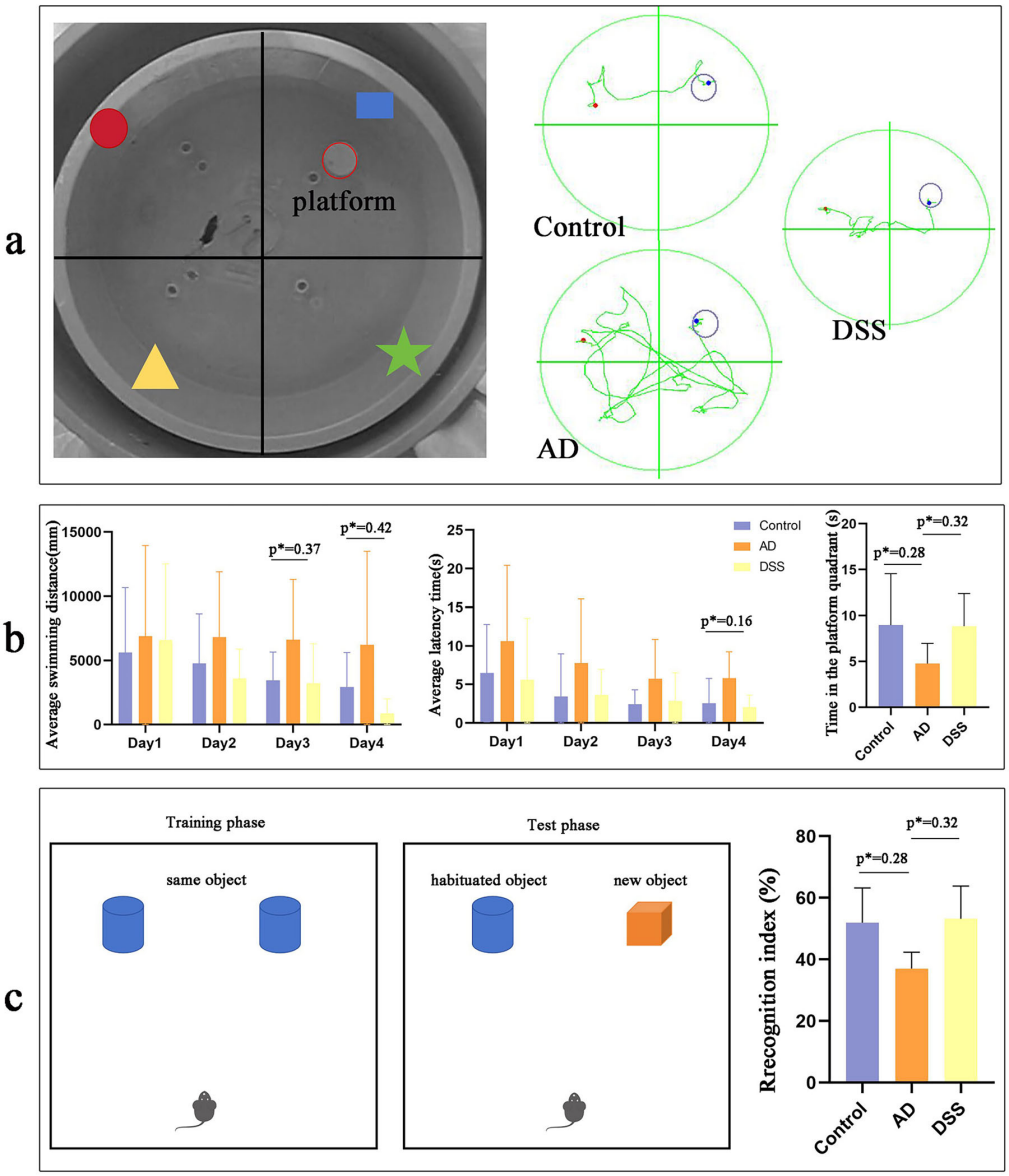
Behavioral experiment. (a) Morris water maze (MWM) layout and mouse swimming tracks were recorded during the experiment. (b) Average swimming distance, average latency time, and time in platform quadrant in MWM. (c) Layout and cognitive index of new object recognition (NOR) (*p < 0.05).

STZ, a neurotoxin, can cause AD‐like cognitive impairment after lateral ventricle injection (Nesrine, Esraa, and Mamdooh 2021). In the positioning navigation experiment of the Morris water maze (Figure 6), with an increase in training time, the average swimming distance and average latency of mice in the control and DSS groups continued to decrease, whereas the AD group did not show a similar performance. The average swimming distance in the DSS group was lower than that in the model group on Days 3 and 4 (*p < 0.05), and the average latency in the DSS group was lower than that in the model group on Day 4 (*p < 0.05). After the platform was removed, the residence time in the quadrant where the platform was located decreased in the AD group compared to that in the control group, and the residence time in the DSS group increased compared to that in the AD group (*p < 0.05), suggesting that DSS improved the memory of the mice regarding the location of the platform. In the new object recognition test (Figure 6), the RI of the AD group was lower than that of the control group, whereas that of the DSS group was higher compared to that of the AD group (*p* < 0.05), suggesting that DSS improved the ability of mice to explore new objects.

**FIGURE 7:**
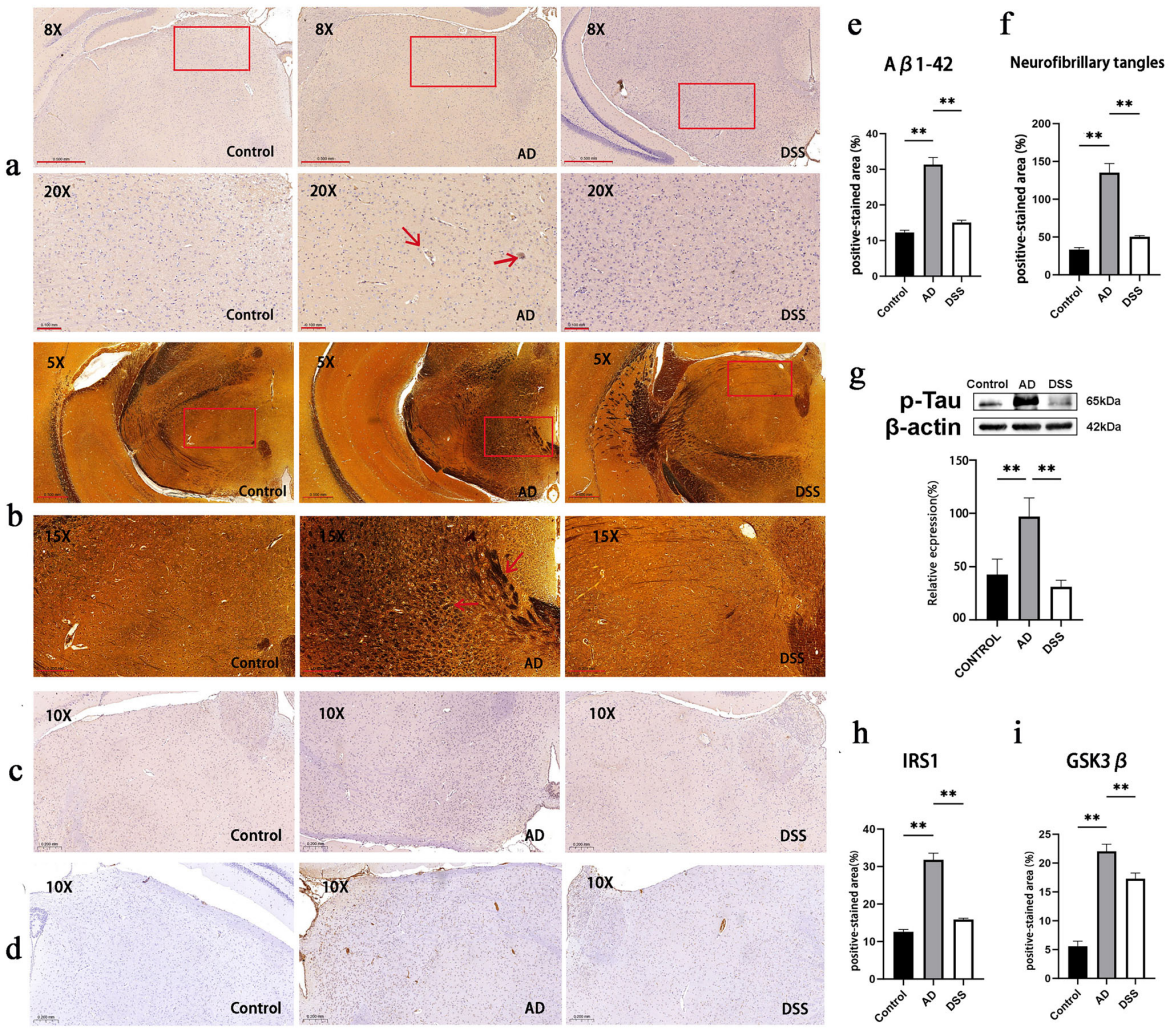
AD‐related pathological changes in the brain. (a, e) Immunohistochemistry analysis showing beta‐amyloid (Aβ) deposition in mouse thalamus (red arrow) (×8, bar = 500 µm; ×20, bar = 100 µm). (b, f) Silver‐coated staining showing neurofibrillary tangles (NFTs) in mouse thalamus (red arrow) (×5, bar = 500 µm; ×15, bar = 200 µm). (c, h) Immunohistochemistry showing positive staining of insulin receptor substrate 1 (IRS1) (×10, bar = 200 µm). (d, i) Immunohistochemistry showing positive staining of GSK3β. (g) Western blot analysis showing relative expression of p‐tau (**p < 0.01).

The thalamus is a key memory‐encoding region that builds episodic memories through clusters of nuclei connected to the hippocampus, temporal lobe, and prefrontal cortex (Weiner et al. 2017). In the present study, immunohistochemical results showed that, compared to the control group, the deposition of Aβ_1–42_ in the thalamus of mice with AD increased and formed age spots (brown mass) (×8, ×20), and DSS intervention alleviated the deposition of Aβ_1–42_ (Figure 7). Silver plate staining showed that compared to the control group, tau hyperphosphorylation levels in the thalamus of mice with AD increased, resulting in increased NFTs (dark cords and patches) (×5, ×15), and NFTs were relieved after DSS intervention (Figure 7). Western blot results showed that the relative expression of p‐tau in the AD group was lower than that in the control group, and DSS treatment reversed this trend (Figure 7).

Bilateral injection of STZ into the lateral ventricles can result in pathological changes in central glucose metabolism, which may be the cause of AD cognitive impairment and an important pathological change in SAD (de Paula Faria et al. 2022). In terms of glucose metabolism, IRS1 and GSK3β are key targets in the insulin signaling pathway. To verify the pathological properties of this model related to central glucose metabolism in AD, we used immunohistochemistry. IRS1‐positive staining increased in the AD group compared to that in the control group, and positive staining decreased after DSS intervention (Figure 7). The area of GSK3β‐positive staining in the AD group was increased compared to that in the control group, and DSS intervention decreased its expression (Figure 7). This suggests that in addition to cognitive damage, this animal model of AD also reflects the disruption of the central insulin signaling pathway, and DSS intervention may affect the expression of IRS1 and GSK3β in the central glucose metabolism pathway.

### 3.3 DSS Alleviates Pathological Changes of the Colon in Mice With AD

**FIGURE 8:**
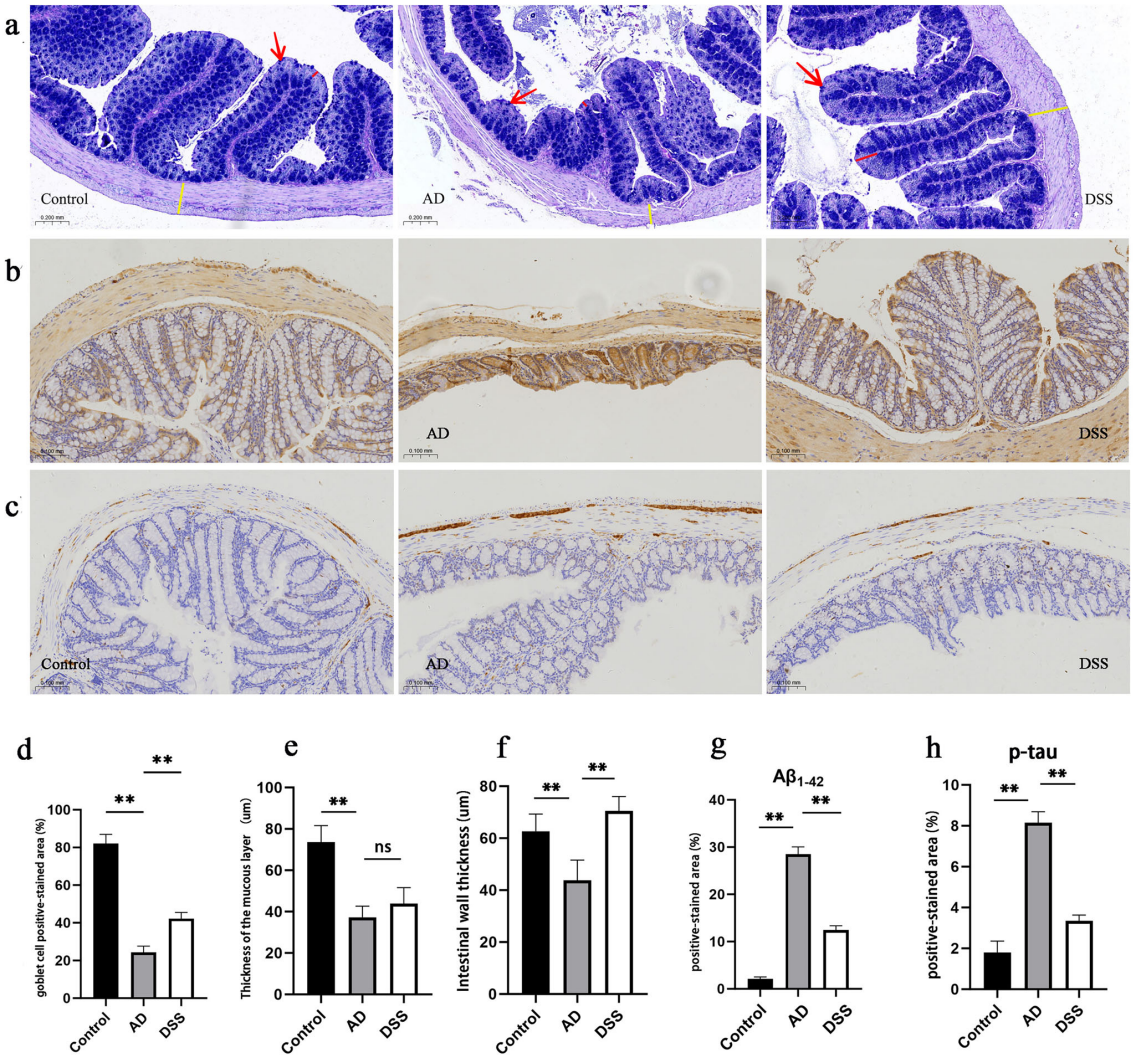
AB‐PAS staining and immunohistochemical results of colon. (a) AB‐PAS staining. Goblet cells are stained blue (red arrow), mucus thickness (red line), and intestinal wall thickness (yellow line). (b, g) Immunohistochemistry showing Aβ_1–42_‐positive staining. (c, h) Immunohistochemistry showing p‐tau‐positive staining (**p < 0.01).

Based on the bidirectional regulation of the gut–brain axis, the deposition of pathological products and disruption of insulin signaling in the AD brain are mutually aggravated by the disruption of intestinal homeostasis (Hallie, Savanna, and Frank 2022). AB‐PAS staining showed (Figure 8) that the colon goblet cells of the mice in the control group had more blue–violet staining (red arrow), high mucus thickness (red line), clear crypt structure, high villus height, and thick intestinal wall (yellow line), whereas the colon goblet cells of the mice in the AD group had decreased (red arrow), thin mucus thickness (red line), and unclear crypt structure. The villus height was shortened, intestinal wall thickness was reduced (yellow line), and the above pathological changes (except for mucous layer thickness) were alleviated after DSS intervention (**p < 0.01) (Figure 8). These changes in the intestinal barrier structure suggest that DSS improves intestinal homeostasis in AD.

In addition to the redundancy of Aβ and tau in the brain, the same pathological products are also deposited in the AD intestine, which may predate the discovery in the brain (Minter et al. 2016; Xia et al. 2022). Immunohistochemical results showed that the area of Aβ_1–42_‐positive staining (brown) in the colon of mice with AD increased compared to the control group, and the area of positive staining decreased after DSS intervention (Figure 8). The area of p‐tau‐positive staining (brown) in the colon of mice with AD increased compared to that in the control group and similarly decreased after DSS intervention (Figure 8). These results suggest that DSS can alleviate Aβ deposition and tau hyperphosphorylation in the colon of mice with AD.

### 3.4 DSS Improves Neuroultrastructural Damage in Mice With AD

**FIGURE 9:**
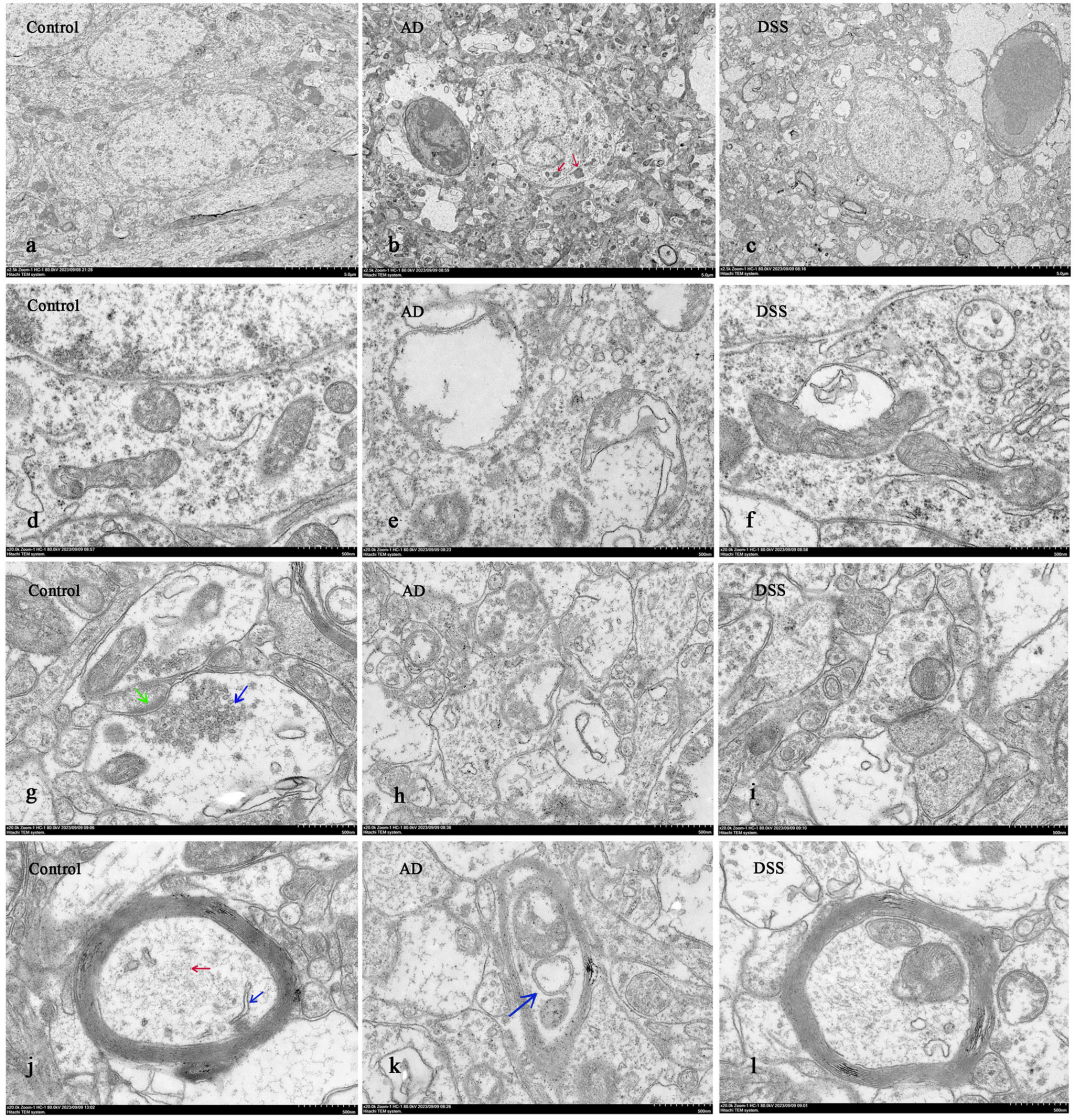
Nerve ultrastructure observed by transmission electron microscopy. (a–c) Neuron morphology and lipofuscin deposition (red arrow); (d–f) mitochondrial morphology; (g–i) synapse; and (j–l) myelin and inner microtubules, microfilaments, and mitochondria (×20.0k).

Disturbances in intestinal homeostasis can interfere with the stability of the central nervous system through complex immune‐related endocrine and metabolic pathways (Collins and Bercik 2009; Fülling, Dinan, and Cryan 2019; Roager and Licht 2018). Transmission electron microscopy showed that the cell bodies of neurons in the control group were filled with large and round nuclei (Figure 9), whereas the cell bodies of neurons in the AD group were irregular and lipofuscin was deposited in the cytoplasm (red arrow), which is an important product of aging (Figure 9). The mitochondria in the control group were abundant and clearly structured (Figure 9), whereas those in the AD group were swollen, the matrix became shallow, and the ridges disappeared (Figure 9). The control group had abundant synaptic vesicles (blue arrows) and synaptic densification (green arrows), which are key components of the neurotransmitter release process, which were reduced in the AD group (Figure 9). In the control group, the myelin sheath was complete and the lamellar structure was clear (Figure 9), with abundant microtubules (red arrow) and mitochondria (blue arrow). In the AD group, the myelin lamellar structure was disintegrated (Figure 9), and the mitochondria were enlarged into vacuoles (blue arrow). All the above pathogenic neuro‐ultrastructural changes were alleviated after DSS intervention (Figure 9).

## 4 Discussion

Intestinal homeostasis is a state of dynamic equilibrium formed by the interaction of the intestinal environment (including the intestinal microbiota, nutrients, and metabolites), intestinal mucosa, and immune barrier. Other alterations in the gut microbiome have been shown to be associated with AD (Ling et al. 2020). In the current study, the AD mouse model was prepared by bilateral lateral ventricle injection of STZ, which not only showed deposition of typical pathological markers and an abnormal central glucose metabolism pathway but also disruption of intestinal homeostasis (microbiota and intestinal barrier), similar to previous studies (Amy 2022; Novotny et al. 2023). The results of 16S sequencing showed that the abundance ratio and diversity of gut microbes in the three groups of mice were different. In terms of microbial composition, the core communities in the gut were mainly Firmicutes and Bacteroidetes, whereas there were many new OTU sequences in the AD and DSS groups, which may potentially lead to the enrichment of microbial functions in the gut, thus changing the composition of the host intestinal flora and affecting the metabolic functions of microorganisms. The results of metagenomeSeq difference analysis showed that the AD host selected beneficial microorganisms to coexist, whereas DSS intervention accelerated host recovery, and the rapid selection of beneficial microorganisms (such as anti‐inflammatory clostridiales) led to the improvement of host intestinal microorganisms, suggesting that DSS promoted the recovery of intestinal microbial function by changing the composition of microorganisms.

Intestinal homeostasis is closely related to the intestinal barrier. The intestinal epithelium not only participates in intestinal microbial defense but also affects digestion and absorption, the mucosal barrier, and immune regulation (Allaire et al. 2018). Goblet cells, as intestinal epithelial cells, are distributed between and within crypts, and their main function (especially the former) is to synthesize and secrete mucus, which not only shields microorganisms but also provides channels for nutrient absorption (Nyström et al. 2021). The secreted products play a role in regulating T cell immunity and promoting mucosal epithelial repair after injury (Soderholm and Pedicord 2019). Goblet cells also deliver soluble antigens from the intestinal lumen to dendritic cells, thereby participating in secondary immunity. In addition to the intestinal epithelium, the mucosal layer is a component of the mucosal barrier. The complex microbial communities in the gut work together to shape the intestinal immune response and inhibit pathogen colonization, playing an important role in immune defense mechanisms in healthy and diseased humans (Sharpen et al. 2022). The function of the mucosal layer is to isolate the luminal contents from the intestinal epithelium; prevent the microbiome and large molecules from contacting the epithelial cells while allowing small molecules to pass through; and prevent the epithelium from contacting acids, digestive enzymes, and microorganisms in the intestinal lumen (Schlechte et al. 2023).

The disruption of intestinal homeostasis is closely related to the pathological features of AD, especially the deposition of Aβ. A previous study observed intestinal dyshomeostasis in the gut of tg2576 mice, which predates Aβ accumulation in the brain (Honarpisheh et al. 2020). The 5‐lipoxygenase produced by the dysregulation of the gut microbiota can also mediate the phosphorylation of the tau protein (Mathewson et al. 2016). Previous studies have found that the expression levels of Aβ in the gut of patients with AD are increased compared to those of non‐AD patients (Cattaneo et al. 2017). Some researchers believe that Aβ accumulates in the intestinal nervous system earlier than the brain and that Aβ migrates through the lower mucosa after damaging the intestinal immune barrier and affects the cholinergic nervous system of the intestine (Donald and Finlay 2023). Using in vivo imaging techniques and tracers, the researchers observed Aβ deposits in the cranial nerves 1 year after the appearance of Aβ deposits in the colon (C. S. Kim et al. 2021). Similar to these reports, we observed significant Aβ deposition and elevated tau phosphorylation levels in the colon of mice with AD, but unfortunately, we were unable to verify the sequence of observations at different time points, which to some extent supports the conclusions of some previous studies. Interventions based on the gut microbiota have also been recognized in several studies. For example, phenolic acid, a polyphenol derivative of gut microbiota, reduces the expression of Aβ oligomers (Wang et al. 2015). In the Aβ‐induced AD model, targeted photobiological regulation can improve learning ability, senile plaque deposition, tau phosphorylation, and the neuroinflammatory response in mice by reversing the imbalance of intestinal flora (Koller et al. 2019). Antibiotics and probiotics have been particularly effective in APP/ps1 mice, which not only reduce the burden of Aβ and alleviate neuronal apoptosis but also correlate with decreased reactive glia around plaques, changes in microglia morphology, and decreased levels of peripheral inflammatory mediators (Minter et al. 2017). In the present study, the Chinese herbal compound DSS with multitarget action was selected, which not only alleviated the age spots and NFTs in the brain AD mice but also played an excellent role in clearing intestinal Aβ and p‐tau deposition, which are related to DSS regulating intestinal microbial composition, protecting the intestinal barrier, and improving intestinal homeostasis.

Intestinal homeostasis affects neural network homeostasis, and our results show that DSS not only improves the intestinal microbiota but also alleviates damage to various neural ultrastructures (including neurons, synapses, and mitochondria) and maintains the stability of the nervous system. A previous study based on 7‐month‐old 5xFAD mice published findings similar to ours in which 5xFAD mice had an increased F/B ratio compared to wild‐type mice, and this gut inflammatory response coincided with synaptic dysfunction in the brain (Ramirez‐Celis et al. 2017). Neural network homeostasis is important for cognitive protection. Abnormal neural network activity may exacerbate the propagation of Aβ and tau in the brain, in which neuronal dysfunction and synaptic damage are the key mediators of the imbalance of neural network activity (Jun et al. 2020). Aβ reduces ATP levels, damages Ca^2+^ homeostasis in the endoplasmic reticulum, and causes swelling of hippocampal synapses and mitochondria (Ramirez‐Celis et al. 2017). We observed a large amount of lipofuscin deposition and structural damage to mitochondria and synapses in AD mouse neurons. The structural disturbance of these key elements in the neural network may be related to an imbalance in the intestinal flora of patients with AD, which is one of the causes of accelerated cognitive dysfunction. Our study also examined changes in myelin structure. The myelin sheath not only ensures the conduction of nerve impulses but also plays a role in the nutrition, plasticity, and metabolism of inner axons (Nickel and Gu 2018). In the present study, DSS intervention repaired the myelin lamellar structure and increased the microtubule skeleton in the myelin sheath. In general, damage to myelin structures is age‐sensitive, especially in patients with AD. Breakdown of the myelin structure leads to reduced axon and ganglion spacing, which not only affects nerve conduction but also increases the brain's susceptibility to ischemia, metabolic abnormalities, and prions. Balanced microtubule dynamics in axons can not only prevent Aβ deposition from damaging synapses but also improve cognition by protecting neuronal homeostasis (Peris et al. 2022).

## 5 Conclusion

In previous studies, evidence supporting the bidirectional relationship between the gut microbiome and AD has mainly been based on transgenic AD models. In our study, an icv‐STZ AD mouse model was used, and the mice showed central Aβ deposition, NFTs, central glucose metabolism abnormalities, and changes in the intestinal flora. In this model, DSS not only alleviated intestinal homeostasis but also improved cognition and maintained nervous system homeostasis, which may be related to the regulatory role of the gut–brain axis in AD cognitive impairment.

## Author Contributions

**Ya‐Han Wang**: writing–review and editing, writing–original draft, funding acquisition. **Peng‐Li Ding**: project administration. **Kai‐Xin Zhang**: project administration. **Xiang‐Qing Xu**: supervision, funding acquisition. **He‐Li**: methodology, supervision.

## Conflicts of Interest

The authors declare no conflicts of interest.

### Peer Review

The peer review history for this article is available at https://publons.com/publon/10.1002/brb3.70110.

## Data Availability

Data will be made available by contacting Ya‐Han Wang.
